# Supplementary material for: A comprehensive study of CYP2E1 and its role in carcass characteristics and chemical lamb meat quality in different Indonesian sheep breeds
Source: PLoS One. 2024 Sep 9;19(9):e0310336. doi: 10.1371/journal.pone.0310336 (PMC11383218; doi:10.1371/journal.pone.0310336)
Supplement: Attachment — Submitted filename: PONE-D-24-10259.docx [file pone.0310336.s005.docx]

The frequencies of the g.50658168 T>C single nucleotide polymorphism (SNP) on the CYP2E1 gene, which is known to affect meat quality, were investigated in Indonesian sheep in this study. Additionally, an association research was carried out involving genotypes of Javanese thin-tail sheep. The introduction section presents a comprehensive assessment of the relevant literature, highlighting the subject's significance. Nonetheless, for the reasons detailed below, it is critical to make specific revisions to the methodology and discussion sections. By the way, the authors clearly outlined the study in the conclusion.

**Major comments:**

L129: What is the definition of concentrate GTO3? Kindly specify in detail, avoiding from abbreviations. Furthermore, there is evidence to suggest that the JTTS oxen used in the slaughterhouse were obtained from multiple farms located in West Java, Indonesia, and were fed in group cages. Nonetheless, the descriptions given are extremely general. It is imperative to specify the types of forage and concentrated feed employed, as well as the duration of their administration.

L159-161: Cooked meat samples are typically employed for Warner-Bratzler Shear Force (WBSF) analysis. Kindly elaborate on the preparatory procedures preceding WBSF. In cases where WBSF was conducted on raw meat, as reported in certain studies, please specify this approach.

L175: Please specify the column utilized for the fatty acid analysis and provide relevant properties. A similar situation is valid for cholesterol, flavor and odor measurements.

L198-200: Which instrument did you employ for the mineral content analysis of the LD muscle, the ICP-OES or the AA spectrophotometer? Please provide specific details.

L219-220: In your study, it was noted that JTTS rams were utilized, with a distribution of CC (n=33), CT (n=58), and TT (n=9) genotypes. Could you elaborate on the rationale and methodology employed for obtaining liver tissue samples from Garut composite sheep rams?

L261-262: In determining the grade of sheep groups, what specific criteria are utilized? Please share extra specific details about your methods.

L520-522: An apparent discrepancy is evident in either the materials and methods or in the discussions: While JTTS rams were indicated to have been used for slaughter in the materials and methods, there appears to be a contradiction in attributing the observed variances to breed differences.

**Minor comments:**

Please verify that abbreviations used throughout the text are appropriately placed.

L25: CYP2E1 appears in numerous places throughout the text, both in italics and in normal form. Each must be italicized for correction.

L26: Please use “g. 50658168” instead of “50658168”.

L28, L39, L148, L429: Please use “fatty acid” instead of “fat acid”.

L60, L61: Please use “grams/capita/year” instead of “grams per capita per year”.

L313, L335: Please ensure that each abbreviation used in the table is accompanied by a corresponding footnote below the table

L340: Please use “fat” instead of “Fats” in Table 4.

L353: Please provide the section where lipid numbers are presented within parentheses.

L386: Please use “potassium (K)” instead of “Kalium (K)”.
