# Supplementary material for: A comprehensive study of CYP2E1 and its role in carcass characteristics and chemical lamb meat quality in different Indonesian sheep breeds
Source: PLoS One. 2024 Sep 9;19(9):e0310336. doi: 10.1371/journal.pone.0310336 (PMC11383218; doi:10.1371/journal.pone.0310336)
Supplement: Attachment — Submitted filename: Response to Reviewers.docx [file pone.0310336.s006.docx]

**Response to Reviewer's Comments**

**Comments from Reviewer 1:**

Comment 1: The manuscript is sound and worthy of publishing, although the research is targeted towards a specific group of researchers.

**Response** 1: Thanks for the consideration and review of our manuscript.

Comment 2: The English needs to be revised.

**Response** 2: We appreciate the reviewer's comments. An English-speaking expert in animal science has checked the manuscript. All changes and corrections are in the revised manuscript with track changes.

Comment 3: However, it is not clear which genotype is desired and why.

**Response** 3: Thanks for the comment. Based on an association study analysis between the CYP2E1 gene and lamb quality, the results showed that the CT genotype had the best value for lamb quality traits: retail cut carcass, physicochemical lamb, unsaturated fatty acid, mineral content, lower saturated fatty acid, cholesterol value, flavor, and odor compounds. We have added this point in the abstract and conclusion.

Comment 4: Tables 2 to 8 describe similar association data, so they could be merged into a single table.

**Response** 4: Thanks for the suggestions. For the analysis results, we have combined tables 3 to 8 into one comprehensive table, which we make in Table 3. Please see line 396 in the revised manuscript with track changes.

Comment 5: Why was mRNA expression performed in tissues with different genotypes, while protein localization was performed in kidney and muscle tissues?

**Response** 5: Thanks for the question. We analyzed mRNA expression based on genotype variations to see the CYP2E1 gene expression. Next, to validate this mRNA expression, we analyzed CYP2E1 protein expression directly in muscle samples to see whether CYP2E1 expression was also present in the sheep muscle samples we used. We validated this protein's expression using positive and negative controls. The positive control was used from kidney tissues because the CYP2E1 protein is widely expressed in kidney tissues (Abdelmegeed *et al.* 2017; Howard *et al.* 2003), while negative controls were used without primary antibodies and replaced with PBS. Please see the revised in lines 269-273 and 311-314.

References :

Abdelmegeed, M. A., Choi, Y., Ha, S.-K., & Song, B.-J. (2017). Cytochrome P450-2E1 is involved in aging-related kidney damage in mice through increased nitroxidative stress. Food and Chemical Toxicology, 109, 48–59. doi:10.1016/j.fct.2017.08.022

Howard L.A., Miksys S., Hoffmann E., Mash D., Tyndale R.F. Brain CYP2E1 is induced by nicotine and ethanol in rat and is higher in smokers and alcoholics. *Br. J. Pharmacol.* 2003;138:1376–1386. doi: 10.1038/sj.bjp.0705146.

Comment 6: Does the lower protein expression in muscle compared to the kidney hold any significance?

**Response** 6: We appreciate the reviewer's comments. The protein expression analysis only validated CYP2E1 protein expression in muscle tissues. We used kidneys as positive controls to test whether the CYP2E1 antibodies and our analysis methods were correct. The previous study reported that the CYP2E1 protein expression is often found in kidney tissues. Therefore, we tested whether CYP2E1 protein was also expressed in our sheep muscle samples.

Comment 7: What is the relationship between the data in Figure 4 and CYP2E1?

**Response** 7: Thanks for the question. Figure 4 shows the HE staining on the sheep muscle tissue. We carried out HE staining to see the morphology of sheep muscles based on the meat quality values, namely pH and tenderness. However, HE staining results (Figure 4) have no direct relationship to CYP2E1.

Comment 8: Additionally, why were liver tissues used for mRNA expression while phenotypes were measured in muscle tissues?

**Response** 8: Thanks for the comment. We analyzed mRNA expression on liver tissue because we referred to Listyarini et al. 2018 and Kubesova et al. 2019, which also used the same samples. Based on previous studies, CYP2E1 expression appeared frequently in liver samples. Gene expression will generally be distributed in all tissues. However, we tested on tissue with high expression values, namely liver tissues, for more accurate results. On the other hand, we measured meat phenotypes in muscle samples to measure meat quality values directly in muscle samples.

References :

Listyarini K, Jakaria, Uddin MJ, Sumantri C, Gunawan A. Association and expression of CYP2A6 and KIF12 genes related to lamb flavor and odor. Trop. Anim. Sci. J. 2018; 41(2): 100-107. <https://doi.org/10.5398/tasj.2018.41.2.100>.

Kubesova A, Stastny K., Faldyna M, Sladek Z, Steinhauserova I, Borilova G, et al. mRNA Expression of CYP2E1, CYP2A19, CYP1A2, HSD3B, SULT1A1, and SULT2A1 genes in surgically castrated, immunologically castrated, entire male and female pigs and correlation with androstenone, skatole, indole, and Improvac-specific antibody levels. CJAS. 2019; 64 (2): 89–97. <https://doi.org/10.17221/159/2018-CJAS>.

**Comments from Reviewer 2**

**Major comments:**

L129: What is the definition of concentrate GTO3? Kindly specify in detail, avoiding from abbreviations. Furthermore, there is evidence to suggest that the JTTS oxen used in the slaughterhouse were obtained from multiple farms located in West Java, Indonesia, and were fed in group cages. Nonetheless, the descriptions given are extremely general. It is imperative to specify the types of forage and concentrated feed employed, as well as the duration of their administration.

**Response**: Thanks for the suggestion. GT-03 concentrate is a type of trade product from Indofeed. All rams were housed in group cages and given king grass forage (*Pennisetum purpureophoides*) ad libitum. In the afternoon, they were supplemented with GT-03 Indofeed concentrate in the amount of 400 g/head/day, while water was given ad libitum. We have added the explanation in the revised manuscript. Please see lines 136-139 in the revised manuscript with track changes.

L159-161: Cooked meat samples are typically employed for Warner-Bratzler Shear Force (WBSF) analysis. Kindly elaborate on the preparatory procedures preceding WBSF. In cases where WBSF was conducted on raw meat, as reported in certain studies, please specify this approach.

**Response**: Thanks for the comments. We have added the specific approach for WBSF analysis. For more explanation, please see the revised manuscript at lines 186-194.

L175: Please specify the column utilized for the fatty acid analysis and provide relevant properties. A similar situation is valid for cholesterol, flavor, and odor measurements.

**Response**: Thanks for the suggestion. We have added a column for fatty acid, cholesterol, flavor, and odor analysis for each procedure. Please see the revised manuscript on lines 207-209 for fatty acid, 226-228 for cholesterol, and flavor odor in lines 238-240.

L198-200: Which instrument did you employ for the mineral content analysis of the LD muscle, the ICP-OES or the AA spectrophotometer? Please provide specific details.

**Response**: We appreciate the reviewer's comments. Mineral content analysis was done using the atomic absorption spectrometry (AAS) method. For the detailed information, please see lines 245-249 in the revised manuscript.

L219-220: In your study, it was noted that JTTS rams were utilized, with a distribution of CC (n=33), CT (n=58), and TT (n=9) genotypes. Could you elaborate on the rationale and methodology employed for obtaining liver tissue samples from Garut composite sheep rams?

**Response**: Thanks for the suggestion. The quantitative real-time PCR analysis was carried out to observe the *CYP2E1* gene expression based on differences in genotype. Liver tissue samples for gene expression analysis were selected from a total of 100 rams (CC (n=33), CT (n=58), and TT (n=9)). A total of 9 samples were selected representing three genotypes, CC, CT, and TT, each genotype consisting of 3 individuals. Three groups of rams were formed based on the TT, CT, and CC genotypes, which had high and low pH and tenderness values. Please see the revised manuscript with track changes for detailed changes (L69-273).

L261-262: In determining the grade of sheep groups, what specific criteria are utilized? Please share extra specific details about your methods.

**Response**: Thanks for the valuable comment and suggestion. The HE staining was carried out to see the morphology of lamb muscle based on the quality value of the meat, which, in this case, we determined based on the pH value and tenderness. We sorted individuals with high and low meat quality scores. We selected three rams representing each group for HE staining analysis. We have added the revision in the manuscript (L276).

L520-522: An apparent discrepancy is evident in either the materials and methods or in the discussions: While JTTS rams were indicated to have been used for slaughter in the materials and methods, there appears to be a contradiction in attributing the observed variances to breed differences.

**Response**: Thanks for the suggestions and comments. We analyzed CYP2E1 gene expression based on variations in the CYP2E1 genotype. We selected nine samples representing three genotypes, CC, CT, and TT, each genotype consisting of 3 individuals. However, we did not find variations in these three genotypes from the same breed, so the nine samples we chose were taken from the JTTS and GCS breeds. The analysis of genotype variations showed that the CT genotype had higher expression values than the CC and TT genotypes. The differences may also influence the higher expression in the sheep breeds. We have corrected the sentence marked in line 591-592.

**Minor comments:**

Please verify that abbreviations used throughout the text are appropriately placed.

**Response**: Thanks for the suggestion. We have corrected the abbreviations we use in the manuscript. For detailed changes, please see the revised manuscript with track changes.

L25: CYP2E1 appears in numerous places throughout the text, both in italics and in normal form. Each must be italicized for correction.

**Response**: We appreciate the reviewer's comments. We have rewritten all CYP2E1 according to the nomenclature requirements (<https://academic.oup.com/molehr/pages/Gene_And_Protein_Nomenclature>). When the text refers to the protein it shall not be italics. We use italics only when we refer to the gene or the mRNA. Please see the revised manuscript with track changes for detailed changes.

L26: Please use “g. 50658168” instead of “50658168”.

**Response**: Thanks for the suggestion. We have corrected the sentence based on the suggestion. Please see the revised manuscript in line 26.

L28, L39, L148, L429: Please use “fatty acid” instead of “fat acid”.

**Response**: Thanks for the suggestion. We have used fatty acids in our manuscript. For detailed changes, please see the revised manuscript with track changes.

L60, L61: Please use “grams/capita/year” instead of “grams per capita per year”.

**Response**: We appreciate the comments. We have rewritten "grams/capita/year." Please see the revised manuscript (L60, L61)

L313, L335: Please ensure that each abbreviation used in the table is accompanied by a corresponding footnote below the table

**Response**: Thanks for the suggestion. We have revised the table according to the reviewer's recommendation. Please see the revised manuscript with track changes for detailed changes (lines 373-374).

L340: Please use “fat” instead of “Fats” in Table 4.

**Response**: Thanks for this point. We have used fat in our table. Please see in Table 3 (L396)

L353: Please provide the section where lipid numbers are presented within parentheses.

**Response**: Thanks for the comment. We have presented the lipid number within parentheses. Please see in Table 3 (L396)

L386: Please use "potassium (K)" instead of "Kalium (K)."

**Response**: Thanks for the suggestion. We have used potassium (K) in our results. Please see the revised manuscript, especially in Table 3 (L396).
