# Supplementary material for: A comprehensive study of CYP2E1 and its role in carcass characteristics and chemical lamb meat quality in different Indonesian sheep breeds
Source: PLoS One. 2024 Sep 9;19(9):e0310336. doi: 10.1371/journal.pone.0310336 (PMC11383218; doi:10.1371/journal.pone.0310336)
Supplement: Attachment — Submitted filename: Response to Reviewers.docx [file pone.0310336.s007.docx]

**Response to Reviewer's Comments**

**Reviewer #1**: The author has responded to all the comments and performed necessary changes in the revised manuscript. The manuscript should be accepted for publication.

**Response**: We are grateful for your positive feedback and your consideration of our manuscript for publication.

**Reviewer #3**: The paper entitled "A comprehensive study of CYP2E1 and its role in carcass characteristics and chemical lamb meat quality in different Indonesian sheep breeds" deals with an interesting topic with aim of evaluating the polymorphism of CYP2E1 gene and its role on the lamb meat quality of 7 Indonesian sheep breeds. Overall, the paper presented high quality of data, however, their discussion is lacking of scientific approach. Moreover, in the material and methods were not clear how much samples and replicates were used for each analyses, which is an important issue to be covered in order to demonstrate the strenghtness of the results obtained. Such an example, the data of polymorphism analysis were not clear on what number of samples have been obtained, on the contrary the real time PCR was performed on three animals for each species treated; I believe that is very low numbers of samples considering the importance of this analysis in the determining the role of the CYP2E1 in meat quality, in this context the main conclusion could not be supported by data. The main criticism is on the discussion which contains a large number of results, including p values, tables and figures references, so it was more a results section than discussion section, therefore, it need to be completely rewritten.

**Response**: We appreciate your comments and suggestions, which reflect your deep understanding of the subject matter. We have explained the samples used in the material and method and have rewritten the discussion section according to your suggestion. In the real-time PCR analysis, we used a total of 9 samples representing three genotypes, CC, CT, and TT, each genotype consisting of three individuals. We acknowledge that the small sample size in our study may not fully represent the overall results. Therefore, we agree with your assessment that further validation is necessary to confirm the effect of CYP2E1 gene expression and polymorphisms in different sheep populations.
